# Inventing a herbal tradition: The complex roots of the current popularity of *Epilobium angustifolium* in Eastern Europe

**DOI:** 10.1016/j.jep.2019.112254

**Published:** 2020-01-30

**Authors:** Renata Sõukand, Giulia Mattalia, Valeria Kolosova, Nataliya Stryamets, Julia Prakofjewa, Olga Belichenko, Natalia Kuznetsova, Sabrina Minuzzi, Liisi Keedus, Baiba Prūse, Andra Simanova, Aleksandra Ippolitova, Raivo Kalle

**Affiliations:** a Ca’ Foscari University of Venice, Via Torino 155, 30172, Mestre, Venice, Italy; b Institute for Linguistic Studies, Russian Academy of Sciences, Tuchkov pereulok 9, 199004, St Petersburg, Russia; c Tallinn University, Narva rd 25, 10120, Tallinn, Estonia; d Institute for Environmental Solutions, "Lidlauks”, Priekuļu parish, LV-4126, Priekuļu county, Latvia; e A.M. Gorky Institute of World Literature of the Russian Academy of Sciences, 25a Povarskaya st, 121069, Moscow, Russia; f Kuldvillane OÜ, Umbusi village, Põltsamaa parish, Jõgeva county, 48026, Estonia; g University of Gastronomic Sciences, Piazza Vittorio Emanuele 9, 12042, Pollenzo, Bra, Cn, Italy

**Keywords:** *Epilobium angustifolium*, Ancient herbals, Eastern Europe, source interpretation, Ethnopharmacology, Traditional medicine, History of medicine

## Abstract

**Ethnopharmacological relevance:**

Currently various scientific and popular sources provide a wide spectrum of ethnopharmacological information on many plants, yet the sources of that information, as well as the information itself, are often not clear, potentially resulting in the erroneous use of plants among lay people or even in official medicine. Our field studies in seven countries on the Eastern edge of Europe have revealed an unusual increase in the medicinal use of *Epilobium angustifolium* L., especially in Estonia, where the majority of uses were specifically related to “men's problems”.

**The aim of the current work is:**

to understand the recent and sudden increase in the interest in the use of *E. angustifolium* in Estonia; to evaluate the extent of documented traditional use of *E. angustifolium* among sources of knowledge considered traditional; to track different sources describing (or attributed as describing) the benefits of *E. angustifolium*; and to detect direct and indirect influences of the written sources on the currently documented local uses of *E. angustifolium* on the Eastern edge of Europe.

**Materials and methods:**

In this study we used a variety of methods: semi-structured interviews with 599 people in 7 countries, historical data analysis and historical ethnopharmacological source analysis. We researched historical and archival sources, and academic and popular literature published on the medicinal use of *E. angustifolium* in the regions of our field sites as well as internationally, paying close attention to the literature that might have directly or indirectly contributed to the popularity of *E. angustifolium* at different times in history.

**Results:**

Our results show that the sudden and recent popularity in the medical use of *E. angustifolium* in Estonia has been caused by local popular authors with academic medical backgrounds, relying simultaneously on “western” and Russian sources. While Russian sources have propagated (partially unpublished) results from the 1930s, “western” sources are scientific insights derived from the popularization of other *Epilobium* species by Austrian herbalist Maria Treben. The information Treben disseminated could have been originated from a previous peak in popularity of *E. angustifolium* in USA in the second half of the 19th century, caused in turn by misinterpretation of ancient herbals. The traditional uses of *E. angustifolium* were related to wounds and skin diseases, fever, pain (headache, sore throat, childbirth), and abdominal-related problems (constipation, stomach ache) and intestinal bleeding. Few more uses were based on the similarity principle. The main theme, however, is the fragmentation of use and its lack of consistency apart from wounds and skin diseases.

**Conclusions:**

Historical ethnobotanical investigations could help to avoid creating repeating waves of popularity of plants that have already been tried for certain diseases and later abandoned as not fully effective. There is, of course, a chance that *E. angustifolium* could also finally be proven to be clinically safe and cost-effective for treating benign prostatic hyperplasia, but this has not yet happened despite recent intensive research. Documented traditional use would suggest investigating the dermatological, intestinal anti-hemorrhagic and pain inhibiting properties of this plant, if any.

## Graphical abstract

**Figure:**
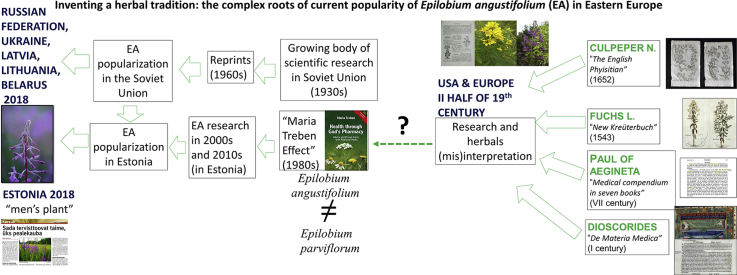

## 1 Introduction

Our field studies in seven countries on the Eastern edge of Europe have revealed an unusual increase in the medicinal use of *Epilobium angustifolium* L. during the lifetime of our interviewees, particularly in Estonia and with the majority of uses being specifically related to “men's problems”. “Men's problems” have become in Estonia a common emic category through popularizing literature and include ailments such as prostate enlargement, prostate cancer, frequent urination, urinary disorders, decrease in sexual potency, the lack of energy due to old age, and so on. While searching for explanations for such a boost in interest and use, we noticed the large extent to which proper historical research is undervalued in current ethnopharmacological scholarship and how important it is for accessing the “traditionality” of a particular plant use in the era of the Internet, where a wealth of historical sources is just a “click away”.

*Epilobium angustifolium* L. (also known as *Chamerion angustifolium* (L.) Holub. and *Chamaenerion angustifolium* (L.) Scop., Onagraceae) has recently become of great interest in scientific research. In November 2015 the plant (along with *Epilobium parviflorum* Schreb.) was assessed by the European Medicines Agency (EMA) which suggested that it has been used in several countries of the European Union for more than 30 years, thus fulfilling the requirements for traditional use “in the following indications: Relief of lower urinary tract symptoms related to benign prostatic hyperplasia, after serious conditions have been excluded by a medical doctor” (Obmann and Länger, 2015; see also EMA, 2019 for the actualized repository for the ensemble of documents of EMA's *Epilobium* monograph). While many older sources refer to it as an ancient medicinal plant, some authors also suggest that the whole genus was “essentially unknown in the pharmacological and pharmacognostical literature until 1980” when it was advocated by Austrian herbalist Maria Treben (1907–1991) (Vitalone and Allkanjari, 2018).

Dal Cero (2016) found in her review on history of the Swiss medicinal flora that *Epilobium* species were documented exclusively in herbals of the modern and contemporary era and that herbalists in Switzerland recommend the infusion of *Epilobium* species to treat urological problems in men. Granica et al. (2014) refer to the earliest sources (such as Native Americans and American herbalists) and also the new interest in the medicinal use of plants belonging to the *Epilobium* family in Europe thanks to Maria Treben. However, as Granica et al. (2014) also notice, Maria Treben points out in her famous book “Health through God's pharmacy” that *Epilobium angustifolium* “has no medicinal properties” and she instead suggests using *Epilobium parviflorum* (Treben, 1980). The growing popularity of *E. parviflorum* in lay use has provoked a new interest among scientists, who, contrary to Maria Treben's suggestion, have also included *E. angustifolium* in their research. One of the reasons for this was specified by Juan et al. (1988), who said that *E*. *angustifolium* was considered one of the two best known species in the *Epilobium* family, the extracts of which “have been used in folk medicine for the treatment of prostate diseases”. Moreover, *E. angustifolium* was recorded as having been sold as *Herba Epilobii* in pharmacies, constituting up to 30% of the market in Austria in the 1980s (Obmann and Länger, 2015), which in itself certifies only that it has been sold, not that it was traditional knowledge. Moreover, there are no traces of folk medicinal uses of *E. angustifolium* in either Italian or German-speaking areas of Europe during the past two centuries (Guarrera, 2006; Hoffmann-Krayer and Bächtold-Stäubli, 1987); yet there are recent records of using *E. angustifolium* roots and flowers for treating diarrhea in Southern Italy (Menale et al., 2006), probably deriving from literary sources.

The development of *E. angustifolium* research has been proceeding quickly; however, within the last five years, all reviews still find the results too preliminary for conclusions. Granica et al. (2014) suggested that as yet both in vitro studies and in vivo clinical research are still too preliminary for drawing conclusions about the efficacy of preparations derived from various *Epilobium* species. Following the general trend of increased interest in its chemical composition among researchers (especially in Russia thanks to the recent growth in popularity of tea made with *E. angustifolium* leaves), Schepetkin and co-authors (2016) reviewed the growing body of literature on the biological properties and potential clinical usefulness of polyphenols derived from the plant. They suggested that the mechanism of the therapeutic properties has yet to be understood and “although fireweed extract and its components appear to be relatively safe, further clinical studies are also clearly necessary to assess potential adverse effects and interactions with other drugs” (Schepetkin et al., 2016). A rather similar idea was further developed by the most recent review discussing the pharmacology and phytochemistry of *Epilobium* species: while acknowledging the large number of preclinical studies on the anti-inflammatory, antioxidant, antitumor, antimicrobial, analgesic, and anti-androgenic effects, the authors stress the need for clinical studies evaluating safety and efficacy, as well as eventually cost-effectiveness in longer terms (Vitalone and Allkanjari, 2018). Meanwhile, the debate among scientists regarding the basic level of the substances contributing to the perceived healing properties of *E. angustifolium* continues. In 1986, research conducted in Austria concluded that flavonoids can be excluded as active components (Hiermann et al., 1986), yet Juan et al. (1988) found the candidate for the anti-inflammatory properties of *E. angustifolium* to be some unknown flavonoid complex. However, research conducted in mice on the anticancer activity of the flavonoid extract of *E. angustifolium* showed not only the absence of antitumor activity, but also high cytotoxicity of the extract, suggesting the closing all research lines in that direction (Polukonova et al., 2017).

The aim of the current work is a) to understand the recent and sudden increase in the interest in the use of *E. angustifolium* in Estonia; b) to evaluate the extent of documented traditional use of *E. angustifolium* among sources of knowledge considered traditional; c) to track different sources describing (or attributed as describing) the virtues of *E. angustifolium*; and d) to detect direct and indirect influences of the written sources on the currently documented local uses of *E. angustifolium* in the borderlands of the Eastern edge of Europe. We argue that the current trend in the use of *E. angustifolium* to treat prostatic hyperplasia is in fact of literary origin and most likely entered the literature by mistake. At the same time, it seems that the plant is repeating the “success” it previously experienced in the second half of the 19th century.

This contribution is part of a study addressing the evolution of local medical practices in highly literate societies as one of the results of the ERC Starting Grant “Ethnobotany of divided generations in the context of centralization”.

## 2 Data and methods

### 2.1 Field study

**Fig. 1:**
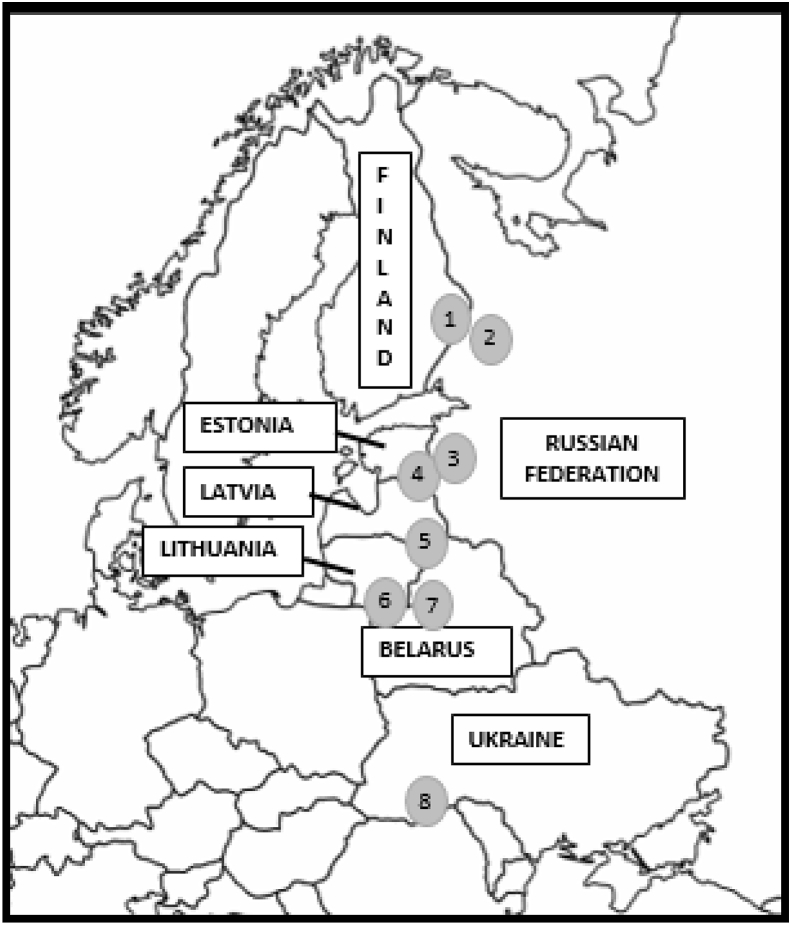
Map of the study area. 1 - North Karelia (Finland); 2 - Republic of Karelia (Russian Federation); 3 - Pskov region (Russian Federation); 4 - Old Võrumaa (Estonia); 5 - Dagda region (Latvia); 6 - Šalčininkai district (Lithuania); 7 - Hrodna region (Belarus); 8 - Chernivtsi region (Ukraine).

*E. angustifolium* is a circumpolar species of the Northern hemisphere, common to large parts of boreal forests and mixed deciduous forests. Growing in open areas and pastures, it is also a well-known pioneer species after forest fires and recently cleared land with sandy-gravelly soils (Mitich, 1999; Pinno et al., 2013). In all of our study areas *E. angustifolium* grows naturally. Field data was collected in the summers of 2017 in Latvia and 2018 in seven different regions located in the border zones of Eastern Europe (Fig. 1, Table 1). While the fieldwork of 2018 was supported by the ERC Starting Grant, the Latvian fieldwork was funded by the Institute for Environmental Solutions. After obtaining oral or written informed consent, we conducted semi-structured interviews with 599 people selected pseudo-randomly. The interviews were conducted in rural areas with a high percentage of forest coverage, a low population density and free access to forests and wild plants. The interviews, which were part of a larger comparative study, lasted from 0.5 to 3 h and covered the wild food and medicinal uses of plants by different ethnic groups inhabiting border regions. *E. angustifolium* was not explicitly emphasized in the interviews, but if mentioned, detailed questions followed in order to identify its precise use. In sum, we collected 11 voucher specimens of *E. angustifolium* and 10 samples of dried plants, of which those originating from EU countries are stored at the Herbarium of Ca’ Foscari University (UVV.EB: SE049, SEDR047, SEDR045, SEDR048, SEDR034, SEDR036, SEDR037, KARDR03, KARDR01, KARDR30, KARDR02, KARDR24a, KARDR04, KAR15, DLGA019, DLGA047, DZULT082, DZULT074, DDZULT23, DDZULT24, DDZULT25, DDZULT35).

**Table 1:** Characteristics of the study sites. For the regions, see Fig. 1.

Region	Languages of the interviewees	Gender (F = female, M = male) composition/mean age
1	Finnish, Karelian	42F, 28M/68
2	Russian, Karelian	58F, 8M/66
3	Russian, Seto	40F, 20M/67
4	Seto, Estonian, Võro, Russian	54F, 41M/64
5	Latvian, Latgalian, Russian	46F, 27M/63
6	Lithuanian, Belarusian, Polish, Russian	53F, 22M/68
7	Belarusian, Lithuanian, Polish	73F, 14M/73
8	Ukrainian, Romanian	55F, 18M/61

### 2.2 Literature research

To better understand the field results we researched historical and archival sources, as well as academic and popular literature, covering the medicinal use of *E. angustifolium* in the regions of our field sites as well as internationally, paying close attention to the literature that might have directly or indirectly contributed to the popularity of *E. angustifolium* at different times throughout history. While we do provide field results, the major part of our work is based on previously published sources; however, it is not in its nature a review, but rather a synthesis and an investigation into the possibilities of interpretation of ethnopharmacological information and an illustration of the diversity of the possible scenarios of interpretation.

We also analyzed the documented ethnographic sources starting with the earliest and most likely authentic, and finishing with more recent and probably already literature-influenced. While we tried to include as many original sources as possible, the analysis of the use of *E. angustifolium* among American Indians is sometimes based on the references and citations provided by the database of Native American Ethnobotany (Moerman, 2003), as it is the best available and most reliable source.

Within the last section of this work, we will look at the historical scientific and popular literature addressing the use of *E. angustifolium*, highlighting the possible sources introducing misinterpretations which led to the attribution of properties that *E. angustifolium* may not actually possess.

## 3 Results and discussion

### 3.1 Field results

The majority of our interviewees had not used *E. angustifolium* as a medicinal plant. Some respondents had heard that it is now a popular herb but had not used it themselves. Among the eight fieldwork sites, *E. angustifolium* was found to be very popular in Estonia, where six, mainly middle-aged, men reported drinking *E. angustifolium* infusion because it was good for well-being or potency, and also against inflammation. Of them, one elderly man (born in 1937) used the tea to treat prostatitis, but he did not feel any effect and stopped. In addition, four women mentioned that they collected the plant to make the tea for their male relatives or/and recommended its use to other male acquaintances as it was “good for health”, for potency, and to treat prostatitis or inflammation. One woman reported its use to alleviate headache, as suggested by her friend from St Petersburg. Notably, the reported sources of influence were various, such as the Internet, books, alternative therapists, and authorities on TV, and all the uses were of recent origin.

In Latvia one man reported the use of *E. angustifolium* tea as “good for health”, whereas one woman reported its use against women's diseases (incl. menstrual pain). Another respondent recalled several uses such as vesical cleaning, cold remedy, immune boosting, alleviate headache. In addition, two women reported the general healthiness of the tea and another woman referred to strengthening of the organism. Its use in treating women's diseases was claimed to have come from family tradition and having been used in the past, whereas other uses were recently learned from either books or the media.

In Ukraine, three people reported diverse uses. A man mentioned that he used tea made from the dried leaves and flowers of *E. angustifolium* to treat inflammation of the duodenum, assuring the researcher that it helped him. Likewise, a woman used tea from *E. angustifolium* for cleansing the blood and as a healthy drink. She referred to the *E. angustifolium* tea as a drink “for strong health”. Another woman used tea prepared from the flowers and leaves to treat her child's cough. She explained that it had a positive effect on the bronchial system and, besides, tea made from flowers has a nice taste and color.

In Lithuania one middle-aged woman claimed she (and her mother) used fermented flowers to treat the prostate problems of her relatives. Another female respondent said that *E. angustifolium* should induce relaxation and sedation, while her husband and her son did not feel such an effect. A Lithuanian-speaking woman in Belarus reported using the plant against inflammation two years ago.

In the Pechory region of the Russian Federation, one woman reported the use of tea made from *E. angustifolium* leaves or flowers as a tranquilizer, another to alleviate headache, and a third against heart disease. In Russian Karelia, one man reported that he treated someone with *E. angustifolium* tea as a tranquilizer and anticonvulsant and claimed getting the desired effect. In Finland older woman said that the tea of *E. angustifolium* has tranquilizing effect.

Notably, almost all medicinal uses were of recent origin, within the last 10 years. Only the use in Latvia to treat women's diseases, originating from the mother, was presented as traditional and old.

### 3.2 The importance of authority and their sources

While in all the other research sites the use of *E. angustifolium* was quite diverse and did not form any meaningful pattern, the results from Estonia are peculiar, not only because it is intensive and well-targeted compared with other sites, but also because it is a good example of both the incorporation of influences from the East and West and the popularization of science. In this case, a clear impact of authority can be detected. The anti-inflammatory use of *E. angustifolium* was first introduced into Estonian through popular print in a small, hiker-oriented brochure in 1991 (Põder, 1991). This was soon followed by the most popular herbal advocate in Estonia at that time, academic doctor Aili Paju (Paju, 1995; Paju, 1996, and many more), who relied on (and referred to) a book in Russian (Minaeva, 1991). *E. angustifolium* became an “official” medicinal plant in Estonia in 1998; however, products containing *E. angustifolium* are not part of a drug or drug-like preparation and thus can be sold outside of pharmacies (Regulation No. 7 of the Minister of Social Affairs in 1998 (RTL, 1998)). After that, widespread promotion began and many authors described *E. angustifolium* as “useful against 99 diseases” (for example Värva, 2004; Niiberg, 2012).

In addition to the sources in Russian, Aili Paju developed the German-language “line” (Maria Treben's book was translated into Estonian in 1991 (Treben, 1991)) about the treatment of prostatitis, which was not present in the Russian-language literature. Aili Paju's writings and appearances on radio and television played a major role in spreading awareness of *E. angustifolium* in Estonia. For example, on March 12, 2006, Paju participated in a broadcast directed at retired people on national TV in which she invited people to send her stories about their personal experiences with *E. angustifolium*. She received numerous letters describing experiences of treating prostate problems, headache, cancer, wounds and skin diseases and also about experiencing the sedative effect of *E. angustifolium*. The majority of people described how they started using the plant for food and treatment only after reading Paju's books and that they were not familiar with this plant before (Tarto and Tammsaar, 2008); letters are stored in the Estonian Folklore Archives in Tartu (EFA I collections, v. 121)).

The suggestions to use *E. angustifolium* against prostatitis were also supported by another representative of academic medicine, professor of pharmacognosy at Tartu University, Ain Raal. In his popular books he states: “Flavonoids give the drug an antitumor, anti-inflammatory and antimicrobial effect” (Raal, 2010). Following his research on the phenolic compounds of *E. angustifolium* (Jürgenson et al., 2012), Raal started to promote it as equal to *E. parviflorum*, which at the time was very highly valued popularly for the treatment of prostatitis, based on Maria Treben's book. Maria Treben wrote that she learned the use of *Epilobium* species with small flowers from her mother, who in turn learned from a neighbor interested in the plants. The neighbor warned specifically against the use of species with big flowers, as their effect was thought to be the opposite of that intended. In the first Maria Treben book officially published in Estonia, there are reports of several miraculous cases of healing people suffering from bladder and abdominal cancer (Treben, 1991). In an interview to the widely read “Eesti Loodus” [Estonian Nature], the country's most popular and long-standing popular science journal, Raal stresses that exclusion of *E. angustifolim* from the list of other suggested *Epilobium* species could have been motivated by Treben's religiousness and unwillingness to use a plant with a local name related to the Devil, although he does not specify the name to which he was referring (Raal, 2017). Ain Raal has been lecturing on the use of *E. angustifolium* for almost a decade, and this might have contributed to the widespread perception of *E. angustifolium* as a “men's medicine” in the summer of 2018 on the Estonian side of Setumaa, while such uses were not recorded on the Russian side. Both Ain Raal and Aili Paju are well-known, trusted and respected specialists among lay people and their suggestions are often amplified by the media.

The Estonian field results, in particular, contrast with those of Finland, where only one use (tranquilizing effect) was mentioned by an enthusiast fermenting *E. angustifolium* tea. At the same time *E. angustifolium* has been popularized by two authoritative sources, earlier by professor of agriculture Toivo Rautavaara (author of numerous books on wild food plants) and today by professor of botany Sinikka Piippo (author of a compendium of herbal folk medicine). For example, Rautavaara explained the exact recipe for making an infusion from the leaves of *E. angustifolium* and says that it is used as a medicine against stomach problems, and in Germany it is used to treat inflammation of the kidneys and urinary tract (Tolvanen, 1995). Piippo (2004) refers to Indian tribes, listing the following uses: to treat cough, tuberculosis, sore throat, skin diseases, various stomach problems, kidney and urinary tract diseases, wounds, and swollen knees, as well as to protect the skin from cold and as a general medicine, among others, including prostatitis.

Medical modernization has always been a contested and convoluted process (Foucault, 1973; Rose, 2007; Scambler, 2002), not least so in the case of recent “modernizers” (Anderson, 2014). Its nonlinear dynamic is highlighted by the rise of discourses and practices of alternative and complementary medicine that themselves emerge and develop within the context of wider societal, cultural and political concerns, and then in turn contribute to these overlapping agendas. In Western societies, the significant increase in the interest in and use of complementary medicine is most commonly placed in the context of postmodern pluralist consumerism and commodification, various social protest movements, rise in discretionary income, as well as decline of civic culture and retreat to private concerns (Cant and Sharma, 2000; Lee-Treweek and Oerton, 2003; McQuaide, 2005; Saks, 2003; Tovey et al., 2004).

While there is far less sociological research on complementary medicine in post-communist countries, our study suggests that even if some of these contexts of its rise partly coincide with Western society – for example, the opportunities entailed in the commercialization of *E. angustifolium* should not be underestimated in explaining its popularization – there are also significant differences. In Finland, we can clearly see the rise of interest in healthy food, yet it is not the case with *E. angustifolium*, probably as a result of very direct use suggestions. Moreover, healing with plants as such was not supported by official medicine in Finland (and neither of the Finnish authors have a medicinal background) and overall we recorded relatively few medicinal uses of plants during our field study compared to other regions.

The greater popularity and more frequent use of complementary medicine in the former Soviet Union republics is commonly associated with the abrupt deterioration of the national health care system after the collapse of the USSR (Brown and Rusinova, 2002; Yurchenko and Saks, 2006) and the subsequent boom of liberalization of publications on the medicinal use of plants a few years before the collapse (Sõukand and Kalle, 2011); it is remarkable that in some cases, the practice of alternative medicine was suggested by mainstream medical doctors (Brown, 2008; Iarskaia-Smirnova and Romanov, 2008). In the case of Estonia, where academic medicine is well advanced, doctor of medicine Aili Paju and professor of pharmacy Ain Raal were, and still are, serving as its mediators at the popular level, following the practice of the collection and official use of medicinal plants (Raal and Sõukand, 2005). Moreover, in Estonia there existed a little-researched unofficial interconnection between academic and popular medicine during the Soviet Union, in which some healers were accepting patients only recommended to them by an academic doctor (Kõiva, 2014).

### 3.3 Known early ethnographic records (before WWII)

During in the 16th century the German scholar Valerius Cordus mentioned a ritual medicinal use of *E. angustifolium*: the aerial parts of a plant that seems to be *E. angustifolium* were worn as “necklaces” for alleviating toothache (Hoffmann-Krayer and Bächtold-Stäubli, 1987). Yet the oldest reliable reports on the use of *E. angustifolium* for medicinal purposes originate from Kamchatka, where early researchers noted that a fermented drink, called *suslo*, made from *E. angustifolium* was used to relieve all sorts of pain (e.g. sore throat), and women drank it for easing severe labor pains; the navel of the newborn was treated with the crunched leaves, which were also used to treat skin diseases (Krašeninnikov, 1755; Steller, 1774). A manuscript written in the 19th century on Estonian local plant uses reports that *E. angustifolium* is named *Solika rohhe* [*Ascarididae* herb] and its seed pods (which looks similar to *Ascarididae*) are given to children when they have stomach worms (Rosenplänter, 1831). An ethnographic record originating from the Perm Governorate of the Russian Empire reports the use of the rarely found white-flowered variety of *E. angustifolium* which was considered supernatural, and a decoction of the flowering plant was used to treat “red and white hernia in women”, epilepsy, headache, unhealthy dryness of the body (*khudoba*) and seizures in children and pregnant women (*rodymchyk*); the last two emic disease names and uses were cited as deriving from a source from 1804 (Krylov, 1876).

Other documented medicinal uses originated in the second half of the 19th century in the Russian Empire and refer to the treatment of constipation (Olonets Governorate), headache (Vladimir Governorate), and chilblains, the latter as a dry powder made from the leaves (Nizhnij Novgorod Governorate) (Annenkov, 1878). There are also records on the use of a decoction against shaking fever (Kazan Governorate) (Krylov, 1882). Later, ethnographic records show that Russians in Siberia used it to alleviate headache (Irkutsk Governorate) (Vinogradov, 1915) and the decoction was drunk to treat scrofula and used to wash pus from the eyes when aching, while the leaves and roots are slowly cooked in milk and applied topically in the case of “chest” diseases (Tobolsk Governorate) (Skalozubov, 1913). Yet the majority of such listings lack further details on the mode of application and preparation or even the parts of the plant used. A report on an expedition to the Altai region provides more specific details on its use to treat scrofula: topical application of heated flowers of *E. angustifolium*, its given local name (*kolokolec zemnoj*), and the village where the information was collected (Katanda) (Utkin, 1933). The Altai region is rather close to Tobolsk Governorate (ca 1000 km), where very similar indications of use were recorded earlier by Skalozubov (1913). A recently published analysis of archival data from the end of the 19th – beginning of the 20th century provides the use of an infusion of *E. angustifolium* in case of difficult childbirth (Voronina, 2015). The only reference point for this claim is that the article analyses the Siberian use of wild plants, yet the use is similar to the ones mentioned above and can be of literary origin.

A book on Estonian plant names (Vilbaste, 1993) also provides one text from the beginning of the 20th century from Hiiumaa Island. It states that *E. angustifolium* (*tulimaa-aluste rohi* [reddening skin disease herb]) is used to treat *urticaria* in children; as a pioneer species after forest burn, *E. angustifolium* could have traditionally been perceived as a cure for burned land, which can be interpreted as an analogy for skin burns. A response to a questionnaire distributed by Polish ethnographer Adam Fischer, conducted in Eastern Galicija (now Western Ukraine) in the 1930s, reported the use of *E. angustifolium* against the culture-bound disease “zawianie” (a “sent disease”, lumbago, “e.g. stiff neck”) which was cured by “fumigation” (Kujawska et al., 2015). At about the same time, it was also used in the treatment of wounds (by applying leaves) and ascariasis in children in southern Poland; and in the province of Krakow *E. angustifolium* was used to fumigate cattle (Kujawska et al., 2016).

It is interesting that the reports on the use of *E. angustifolium* to treat *Ascarididae* infection in Estonia and Poland coincide; however, this may be related to the fact that in the early 18th century, after three devastating plague epidemics, the territory of present-day Estonia was re-populated with people coming from the territories of modern-day Poland, which might have also contributed in addition to visual similarity of seed pods.

The earliest data on the medicinal use of *E. angustifolium* by Native Americans derives from the 1920s and until WWII refers almost exclusively to dermatological uses, according to Moerman (2003). Only a single early source, a book by George Bird Grinnell, who not only lived among the Cheyenne Indians but also herborized the plants they used, provides non-dermatological use. Grinnell (1923) states that the plant's name is translated literally as red (blood) root medicine and the infusion of dried and pulverized leaves (and separately roots) are “given to a person who has hemorrhage of the bowels”.

### 3.4 Tibetan medicine and its research in the Soviet Union

Estonian authors popularizing *E. angustifolium* were greatly influenced by respective literature published in Russian. The first popular book (which sold hundreds of thousands of copies) mentioning the use of *E. angustifolium* was published in the 1970s (Minaeva, 1970). Minaeva states: “In folk medicine, *E. angustifolium* is used to treat scrofula and headache; decoction and tincture are taken in case of metabolic disorders, gastrointestinal diseases, gastric ulcer, and inflammation of the ear, throat, and nose. Topically applied powdered herbs heal wounds. In Tibetan medicine, a decoction of herbs is used as an antipyretic, against scrofula and headaches and as a sedative.” (Minaeva, 1970, emphasis ours). Notably, Tibetan medicine is mentioned in 30 plant chapters, in which she refers to many works by Varlakov and Utkin, who worked on mapping natural resources in Buddhist regions close to the Mongolian border. In one of the chapters, there is the following passage about *E. angustifolium*: “*Sonnyj koren* [sleep root] …”. Decoction is drunk against headache. It is considered sleep-inducing. In addition it is used against gonorrhea and syphilis. Found alkaloid. In leaves found alfa-tannides. Reserves of the resources huge. Found in …” (Varlakov, 1933). The author specifies that the goal of the expedition was to collect wild resources in order to identify new potential alkaloid medicines and for that purpose a rapid in-field chemical analysis was undertaken; and *E. angustifolium* was one of 82 different resources collected. There are references to acquiring information from local people (Varlakov, 1933), yet the ethnographic reliability of this information is rather questionable, as such types of expeditions were generally designed for collecting plant resources for new medicines due to a deficiency of supplies (Conroy, 1998) without involving specialists in ethnographic research.

It is quite remarkable that in Gammerman and Grom (1976), a book published during the lifetime of Adele Gammerman (1888–1978), the prominent scholar of Tibetan medicine in Russia, there is no mention of *E. angustifolium* or reference to Tibetan medicine for other plants. In a later publication, updated by co-authors, Gammerman et al. (1983) do refer to *E. angustifolium*, using text similar to that of Minaeva (1970). Soon after, Mahov (1986) refers to Tibetan medicine in the context of *E. angustifolium*, but cites only Gammerman et al. (1983), thus continuing to promote the same uses through secondary citation. Notably, a monograph on Tibetan medicinal plants published in Siberia (Aseeva et al., 1985) refers to Gammerman and Semichov (1963) stating that *E. angustifolium* shares its name with some more potent medicinal plants, while at the same time cites a book by Varlakov (1963) published after his death as a reference for the anti-inflammatory and coating properties of the preparation of *E. angustifolium*. In that book, in fact, there are two different articles mentioning *E. angustifolium*. One, a reprint of an article published posthumously in a pharmacy journal in 1946, provides a method for assessing the anti-inflammatory and coating properties of plants, in which *E. angustifolium* is seen as the most promising and thus recommended for clinical studies, to be used in low doses in the case of painful inflammation in the stomach area. The other is also a reprint from an article originally published in 1933, which did not include *E. angustifolium*, yet the new version contained a previously unpublished full list of medicinal plants of Eastern Transbaikalia, in which *E. angustifolium* is listed as used to treat syphilis and gonorrhea (Varlakov, 1963)*.* This book had a wider distribution than the original article and this information entered the herbals; for example, Dudchenko et al. (1989) lists, among others, uses to treat gonorrhea and syphilis as deriving from folk medicine. As *E. angustifolium* was absent from the earlier editions of Minaeva's herbal (first edition published in 1951), there are grounds to suggest that she discovered the works of Varlakov through the latest publication (Varlakov, 1963). Minaeva (1970) did not mention syphilis and gonorrhea, as they were common *taboo* subjects in popular Soviet literature before *perestrojka* (1980s; see also Kulasalu (2013) which discusses the censoring of historic folklore collections in Estonia to exclude all *dirty* words during the early years of Soviet occupation), yet mentions all other uses listed in Varlakov (1933) and the treatment of scrofula referred to by Utkin (1933). An earlier herbal published in Minsk in 1965 and edited by Adele Gammerman has a chapter on *E. angustifolium* in which an enlarged thesis of a dissertation by a student of Gammerman is cited (Nikolaeva, 1964). This chapter refers only to the use of flowering aerial parts, heated in the oven and applied topically to aching areas in the case of respiratory inflammation (Gammerman and Jukevich, 1965) - a use that seems rather authentic as such an external topical application of plants is common in the region and there are no traces of a similar use in earlier books, yet such an external use did not find its way into future herbals.

It seems that the popularization of *E. angustifolium* in the Soviet Union intensified against the backdrop of research on Tibetan medicine, which was scientifically considered beginning in the 1930s, despite the fact that practicing Tibetan medicine in the Russian Empire started in the 17th century along with the introduction of Buddhism (Ajusheeva, 2006). While we know that Varlakov published his primary results in 1933, Palibin (1944) reports that the first analysis was only carried out in 1935 and that “very recently the Chemical Laboratory of the Botanical Institute of the Soviet Academy of Science had discovered that *E. angustifolium* belongs to the vitamin plants, containing a high proportion of C-vitamins”. Later, this information was also copied in numerous Soviet books on phytotherapy (e.g. Krylov, 1972; Popov, 1968), and from the end of the 1980s, after the start of mass publication of popular herbals, *E. angustifolium* was present and its medicinal use was promoted in many of them in various combinations of uses.

More recently, Barnaulov, a leading researcher at the Institute of the Human Brain (Russian Academy of Sciences) and the author of many books about various aspects of phytotherapy, has claimed to use *E. angustifolium* (both leaves and flowers) in his practice. He adds *E. angustifolium* to mixtures with a sedative effect, for treating patients with neurosis, hypertension, angina pectoris, and peptic ulcer disease in order to mitigate the effects of psychogenic trauma; he has also published a recipe of this sedative herbal mixture (Barnaulov, 2007).

At the present time *E. angustifolium* is not included in either the State Register of Medicines (State Register, 2019; see also, Shikov et al., 2014) or the State Pharmacopoeia of the Russian Federation (State Pharmacopoeia, 2018), as it was not included in earlier version of the official pharmacopoeia during either the Soviet Union or the Russian Empire (Kukes, 1999).

### 3.5 The North American wave of popularity: second half of the 19th century

*E. angustifolium* was not mentioned in any of the early American herbals published in the first half of the 19th century (for example, Griffith, 1847). However, a breakthrough occurred shortly thereafter, and the plant began to be recommended for a number of diseases. For example, by 1854, it had already been written: “*Properties and Uses.* — Tonic, astringent, demulcent, and emollient. An infusion of the leaves will be found beneficial in chronic diarrhea, dysentery, leucorrhea, menorrhagia, and uterine hemorrhage; and forms an excellent local application for ophthalmia, ulcerations of the mouth and throat, and leucorrhea. The leaves in poultice are a valuable remedy for foul and indolent ulcers. Dose of the infusion from two to four fluid ounces, three or four times a day” (King, 1854). In 1855, American herbalist Dr. Daniel Smith suggested syrup of *E. angustifolium* against dysentery (Smith, 1855).

In 1878, the American pharmacist Biddle wrote that a local physician Dr H. A. Smith had used the roots of *E. angustifolium* treated with water for the cure of aphthae, stressing its “demulcent and astringent properties” (Biddle, 1877). The prominence of *E. angustifolium* as an herbal remedy in this market at that time is also evidenced by the fact that attempts were made to fraudulently substitute its leaves with those of *Salix humilis* (McIntyre, 1876). Within a short period of time, different authors listed nearly all possible applications of *E. angustifolium*. For example, “It seems to have a special affinity to unite with and destroy the microorganisms of diarrhea and dysentery. Its sphere of action is best exhibited upon all organs in the genitourinary organs, hence it is valuable in catarrh of the vagina, bladder, prostate” (Buchanan, 1891). Likewise, “*Epilobium* has proven itself a mild tonic and astringent, quite useful in slight types of diarrhea and dysentery attended with colic, cramps in the stomach, and light typhoid abdominal symptoms. In irritation of the intestinal canal, followed by diarrhea and some tympanitis, it has often proved quite beneficial in the hands of our eclectic physicians” (Millspaugh, 1887).

The ardent support for its use in treating diarrhea is understandable in the context of the belief in the astringent properties of the plant; yet the ethnographic records of its use to alleviate constipation in Olonets Governorate (Annenkov, 1878) and as a laxative (old dried leaves) among the Yup'ik on Nelson Island (Alaska) (Ager and Ager, 1980) are more in agreement with the following reference published in 1887. That particular book of medicine warns against excessive use of the plant, as symptoms include “salivation, loose stools, red urine and chills, followed by feverishness and general aching throughout the body … No analysis of this plant has so far been made. It contains, however, tannin and gallic acid, besides the usual plant constituents” (Millspaugh, 1887). At the same time, Johnson (1884) lists all the beneficial properties from King (1854) and adds: “If the plant be really so valuable as the above statement would indicate, it is rather remarkable that its virtues are not better known and appreciated” (Johnson, 1884).

Nevertheless, in 1897 one can still find *E. angustifolium* among the “natural order drugs” of the USA repeating the same wording (“demulcent and astringent”) and with the dose varying from 30 to 60 gr (Sayer, 1897). But it was no longer an official medicinal herb in the USA by beginning of the 20th century (Henkel, 1906) nor mentioned at the beginning of the 20th century as a significant natural beneficial plant (Saunders, 1920). The official list of herbs in the UK in the 1930s specifies the same uses but also adds the treatment of “whooping cough, hiccough and asthma” (Grieve, [1930] 2013).

### 3.6 Ghost data ascribed to American Indians

Native American uses recorded after WWII still largely refer to the treatment of wounds and skin problems, as well as its use as a laxative or against constipation and other stomach related problems, as in earlier reports, but among these we can also find uses to treat cough and tuberculosis; however, one record suggests that the infusion of plants is considered poisonous (Moerman, 2003). Even by the beginning of the 19th century people were warned repeatedly against drinking *E. angustifolium* tea because it was believed that it was a narcotic “and the Russians are intoxicated with the infusion of it” (Trommsdorff, 1812). This perception might have come from misinterpreting the impact of *E. angustifolium* on hallucinations caused by the use of *E. angustifolium* combined with *Amanita muscaria* (as described by Steller (1774)). It has also been alleged that the fake tea (made from *E. angustifolium* leaves) causes fatigue and numbness of the limbs (Anonymus, 1875). Similar warnings are present in the English literature even now, where it has been said “an infusion of the plant produces a narcotic” (Lewin, 1998) or “stupefying effect” (Grieve, 2013).

In particular, a large number of uses of *E. angustifolium* were recorded among the Iroquois (northeastern Native Americans) by James William Herrick in his doctoral dissertation in 1977 and later published in book form in 1995. According to Moerman (2003), Herrick's dissertation describes the following uses of *E. angustifolium*: “compound of an infusion of roots taken for kidneys or for male urination problems” or against “burning urination”, “infusion of bark (sic!) applied as poultice for pain anywhere in the body”, “decoction of roots taken for internal injuries from lifting”, “poultice of smashed roots applied to swollen knees”, “compound infusion of twigs (sic!) and roots taken as a panacea for pain” and “compound decoction of plants and a doll used for black magic”. Even as mentioning the use of bark (unless the bark of the roots was the intention) and twigs (of a herbaceous plant) cautions us against the reliability of the report, there are a few other questionable details which we can observe in (Herrick, 1995): all listed names of *E. angustifolium* are clearly of European origin (“Willow-herb, Fireweed, Wickup”; p. 174), and this plant is not a part of black magic, but rather the cure for the harm inflicted with such magic (p. 167). While the small discrepancy between the two sources may be due to differences between the dissertation and the later published book, the use related to urination problems seems to clearly be of literary origin from 19th century American herbals.

A recent publication also introduces confusion, probably through the use of inaccurate sources, into earlier American Indian use of *E. angustifolium,* stating: “*Epilobium angustifolium* was called ‘slippery root’ by Chippewa Indians in North America and its leaves were used in treatment of bruises. It was claimed to possess tonic, adstringent, demulcent and emollient properties (Bureau of American Ethnology, 1928)” (Granica et al., 2014 [Emphasis ours, spelling unchanged]). The ethnobotany of Chippewa Indians was indeed addressed in 1928 by Frances Densmore (1928), and the part about bruises probably also derives from this source, yet the rest of the text, including the translation of the name, originates from the combination of Smith (1932) and Smith (1933) (Table 2).

**Table 2:** Early ethnographic records from Native Americans.

According to Moerman (2003)	According to original sources	Source, page	Comments
Root used to make a wash for swellings		Smith (1923)	Original not available online
Poultice of moistened, fresh or dried leaves used for bruises or to remove a sliver	Chew the fresh leaves and stalk, apply as a poultice [on bruise]	Densmore (1928), p. 353	Page mismatch – the description corresponds with the next line
Poultice of roasted and mashed root applied to boils	Bella Coola: Root roasted in ashes, mashed between hands or stones, and applied to boils. Southern Carrier: Not used. Gitksan: Not used	Smith (1929), p. 60	
Poultice of pounded root applied to boils and carbuncles	“Great Willow-herb (*Epilobium angustifolium* L.), “o' ca cadji’ bikes” [slippery or soap root] … The Flambeau Ojibwe say that the outer rind of this root lathers in water, and they pound it to make a poultice. This is used to draw out inflammation from a boil or carbuncle. With white men, it is a demulcent, tonic and astringent. It has been used internally for its tonic effect on mucous surfaces and its value in intestinal disorders.”	Smith (1932), p. 376	Emphasis ours
Plant used as a medicine for unspecified ailments	“Fireweed (*Epilobium angustifolium* L.) … “kêgi'nano'kûk” [sharp pointed weed]. While the Forest Potawatomi use this for medicine, its use was not explained. Among the whites (Nickell, 1911) the leaves and the root have both been used for their tonic, astringent, demulcent and emollient properties.”	Smith (1933), p. 66	Emphasis ours

**Fig. 2:**
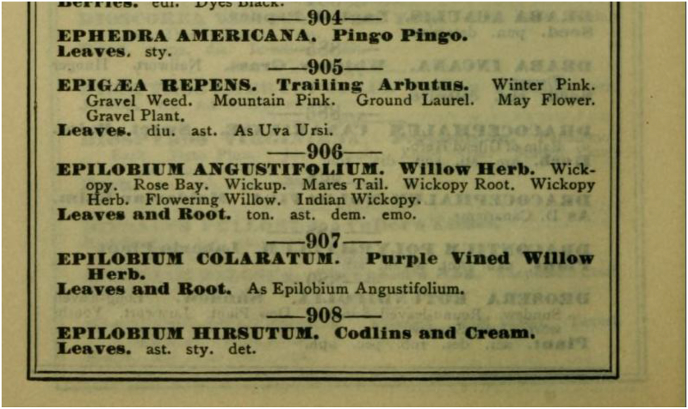
Page 56 of J.M. Nickell's Botanical Ready Reference (Nickell, 1911), in which “white men” uses of *E. angustifolium* are provided.

Smith (1933) directs us to his source, a botanical reference published few decades earlier in Chicago, especially designed for druggists and physicians: “/---/*containing all of the botanical drugs* known up to the present time, giving their medical properties, and all of their *Botanical, Common, Pharmacopæial* and *German Common* (in German) *Names*” (Nickell, 1911). From this single source (Fig. 2) it is not clear, however, if the Indians themselves were referring to those uses as the ones of “white men”; but in comparing different publications of the same author it becomes evident that it is added just for comparison. Nevertheless, subsequent authors present it as a Native American use, and with it creating “ghost data” (*sensu* Svanberg, 2012).

### 3.7 From antiquity to the 19th century: uncertainty about the name allows for a multiplicity of interpretations

We should look for the roots of this phenomenon in the more distant past. Modern written sources clearly referring to the use of *E. angustifolium* seem to be misinterpretations of some ancient works. *E. angustifolium* has a confusing history of naming. Its pre-Linnaean generic name was *Chamaenerion* which according to Sennikov (2011) was first mentioned by Conrad Gessner (1516–1565) in 1561 (Cordus and Gessner, 1561). Later it was referred to by the name *Lysimachia Chamaenerion dicta latifolia* and *Lysimachia Chamaenerion dicta angustifolia* by Gaspard Bauhin (1560–1624) (Bauhin, 1622); yet even before and after that, its nomenclature was constantly changing (Sennikov, 2011). This could have created grounds for erroneous transfer of some uses attributed to other plants from the genus *Lysimachia* or other genera from the Onagraceae family. While the botanical nomenclature changed, the medical nomenclature remained the same for a longer time and was still in use in the second half of the 19th century. For example, the herbals in the German language referred to this name and contained specific indications as early as the beginning of the 19th century: “In ancient times, the root and leaves, *Radix* et *Herba Lysimachiae chamaenerion*, were used as softening, oily and mildly contracting agents” (Winkler, 1840) or “… roots and leaves were otherwise used under the name *Radix et Herba Lysimachiae Chamaenerion* as a softening, healing, slimy, but also mild astringent remedy” (Petermann, 1847). Subsequent authors primarily copied the previous references (for example, Rosenthal, 1861; Karsten, 1883), despite the fact that Reinsch (1844) conducted, for that time, a thorough organoleptic and chemical analysis of the root suggesting that, as it contains tannins, it can be helpful for treating dysentery.

**Fig. 3:**
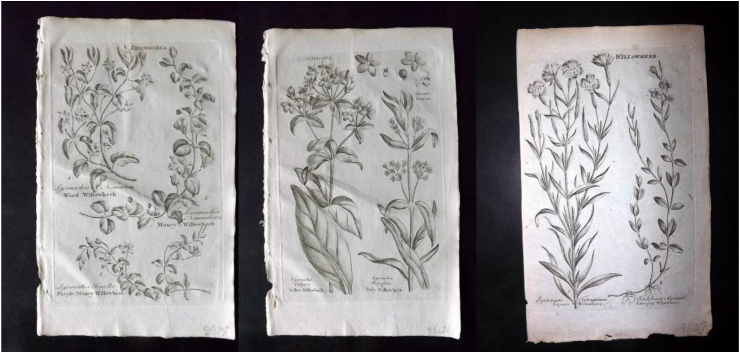
Illustrations of different willow herbs in Hill and Culpeper, 1792 Antique Botanical Print (Hill and Culpeper, 1792). Source: www.albion-prints.com.

Another possible translation trap could have come about through the combination of the name *Lysimachia* and its English equivalent. In 1652, the English botanist Nicholas Culpeper published his famous book which is still considered a classic work today. In it, Culpeper describes *Loos-Strife* or *Willow hearb* as follows: “This Hearb is good to stay all manner of Bleeding at Mouth or Nose or Wounds, and all Fluxes of the Belly, and the bloody Flux, given either to drinke, or taken by Clystor; it stayeth also the abundance of Womens Courses; It is a singular good wound Hearb for green wounds, to stay the bleeding, and quickly to close together the lips of the Wound, if the herb be bruised and the Juyce only applyed: It is often used in Gargles for sore mouthes, as also for the secret parts: the smoke herof being burned driveth away Flyes and Gnats which use in the night-time to molest people inhabiting neere Marshes and the Fenney Countryes.” (Culpeper, 1652: 74). Even though Culpeper does not have a single chapter on *E. angustifolium* or the *Epilobium* family, misinterpretation of Culpeper's words is still occurring today and “willow herb” is translated as *E. angustifolium* with the attribution of all the uses described by Culpeper (see, for example, Bernard, 2018)), despite the fact that the plant description Culpeper provides as well as another English name (loosestrife) clearly refer to the species *Lysimachia* (most likely *L. vulgaris* L.) and none of the later published illustrations resemble *E. angustifolium* (Fig. 3).

**Fig. 4:**
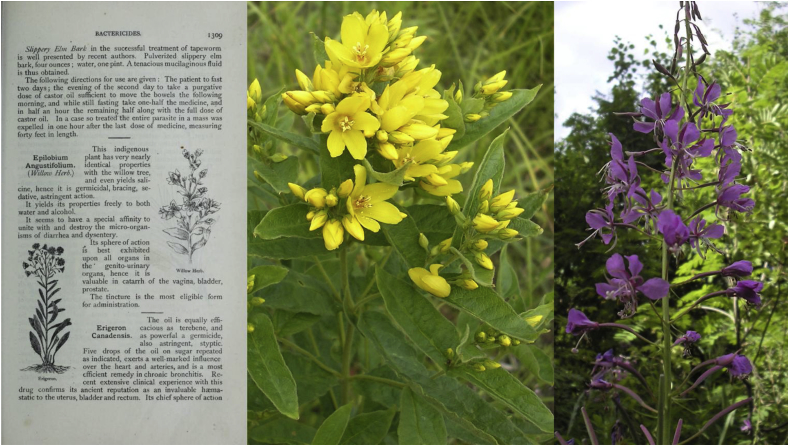
Picture of *Lysimachia* sp. as *E. angustifolium* in Buchanan's Encyclopedia of the Practice of Medicine (Buchanan, 1891); photos of *Lysimachia vulgaris* and *E. angustifolium* for comparison (authors: Raivo Kalle, Renata Sõukand).

The name “willow herb” was at that time a generic term assigned to all plants having leaves resembling those of the willow (Booth, 1835), such as *Lythrum salicaria* (Lythraceae), *Lysimachia vulgaris*, *Lysimachia thyrsiflora* (Primulaceae), *E. angustifolium*, and the whole *Epilobium* genus (Onagraceae). In addition, Griffith (1847) uses the phrase “demulcent and astringent” to describe the medicinal properties of *Lysimachia salicaria* L., which he also calls Purple Willow Herb, so there are many grounds for misinterpretation; see, for example, Fig. 4.

Another similar shared name is St. Antonius herb, referring to its possible use in the treatment of erysipelas. *Herba Antonii* and its relation to *Erysipelas* was previously mentioned by Gesner, who also provides in the same paragraph other familiar names (like *Epilobii*, *Lysimachia* and *Chamaenerion*) (Gessner, 1561). However, throughout the history, the name related to Saint Antonius has been attributed to several taxa, among them *Oenothera biennis* L., which was brought from America in 1614 as an ornamental plant and became known as a medicinal plant about a century later (Van Osta, 2005; Van Osta, 2004). This could have been misleading and uses of *Oenothera biennis* L. may have been transferred to *E. angustifolium*. At the same time, *E. angustifolium* has also been regarded as one of the most plausible candidates for a plant named “plakun - the mother of all plants” in handwritten Russian manuscripts of the 17th-19th centuries (Ippolitova, 2019). Notably, in Switzerland and Western Germany, where flowering aerial parts were an indispensable component of the flower bunches brought to the Church on Assumption Day, *E. angustifolium* was used and known as a protective means against lightning (Hoffmann-Krayer and Bächtold-Stäubli, 1987), while in Poland *E. angustifolium* was similarly used and called *Boże paluszki* or *paluszki Matki Boskiej* (*God's* or *Mary's fingers)* (Łuczaj, 2011).

**Fig. 5:**
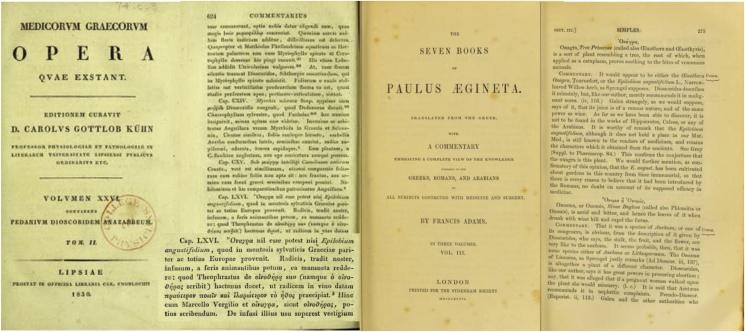
On the left: An example of the attribution of Dioscorides' *Onagra* to *Epilobium angustifolium* (Sprengel, 1829); on the right: a translation of *Onagra* in Paulus Aegineta's “The Seven Books” (Adams, 1844).

Lily Y. Beck, the most current translator of Greek pharmacologist Pedanius Dioscorides (40–90 AD) book *De Materia Medica* [On Medical Material], which is considered the forerunner of all modern pharmacopoeias, relates *E. angustifolium* to *Onagra*/*Oleander*: “Onagra. The oleander that some call Onotheras and others onothuris: it is a tree-like shrub, of a good size, having leaves nearly resembling the almond tree, but broader and resembling the leaves of the white lily. It has large, rose-like flowers, and a long white root that smells like wine when dry. It grows in mountainous places” (Dioscorides, 2017). Yet this association was already known in the 19th century and *E. angustifolium* was regarded as *Onagra* in several translations of *Materia Medica*, but also in the translations of Paulus Aegineta (VII AD) (Fig. 5). Interestingly, both misleading translations were published in London.

In Russia, the linguist Annenkov (1878) also related *E. angustifolium* to *Onagra* and Dioscorides, and this is repeated in modern popular literature (Bugaev, 2010). Such misinterpretations can also be of earlier origin: as early as 1782 an article on *kiprejnik* (among other plants) was published in *Economical magazine*, a supplement to the *Moskovskie vedomosti* newspaper. In it, the reference to taming animals is similar to Dioscorides’ text on *Onagra*: “Doctors attribute it to those herbs, which are used for wounds and all sorts of, that is, bad and decent, abscesses and sores. It is used both internally and externally, stops blood and is used for throat gurgling. Moreover, some foreign writers mention strange things about it, namely: its roots are suitable for taming wild animals and making them tame and humble, which I leave to everyone to judge on his own” (Bolotov, 1782). Compare the modern translation of Dioscorides by Lily Y. Beck: “The infusion of its root has the property of taming wild animals when they drink it.” (Dioscorides, 2017: 294).

**Fig. 6:**
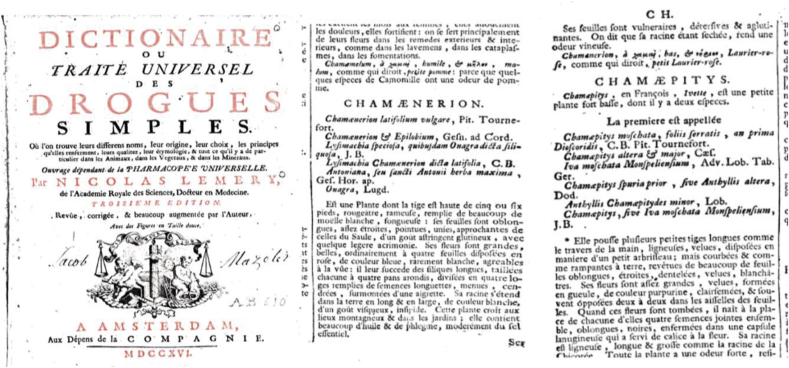
Chapter on *Chamaerion* encompassing several names related to *E. angustifolium* by Lémery (1716).

Yet even earlier, in his dictionary of medicinal plants (Lémery, 1716), compiled by the French alchemist Nicolas Lémery (1645–1715), but published posthumously, includes *Lysimachia Chamaenerion*, *Onagra*, *Epilobium*, *herba Antonii* and some other names within the chapter on *Chamaenerion* The description of the plant resembles that of *E. angustifolium*, but only the use of leaves for the treatment of wounds was described (Fig. 6). While we cannot evaluate the direct influence of this work, it might be one of the very first printed book binding in one chapter *E. angustifolium* and *Onagra*.

The physician Pietro Andrea Mattioli (1501–1578), who philologically reconstructed Dioscorides' *Materia Medica* (first century AD/CE) and was at the time his most reliable Latin translator, did not mention *Epilobium*, yet he commented on Dioscorides' definitions of *Lysimachia* and *Onagr*a (Dioscorides, 2017: 251 and 294). In his commentary on *Onagra*, Mattioli explained that he had never seen it, further added that his friend and botanist Luca Ghini found a plant in the Appennino which might be identified as *Onagra*, but that Ghini did not dare to identify it as *Onagra*, as he had neither checked if the root smelled like wine nor tried to infuse it. In fact, in three illustrated Greek manuscripts of Dioscorides’ work, whose reproductions are now available online (Whipkey et al., 2014), there is only a depiction of *Lysimachia* and no image can be found for *Onagra* or *Epilobium*. Clearly there was no identification of *Onagra* as *Epilobium*, neither according to ancient sources like the illustrated manuscripts nor the experience of a skilled botanist and physician like Pietro Andrea Mattioli.

**Fig. 7:**
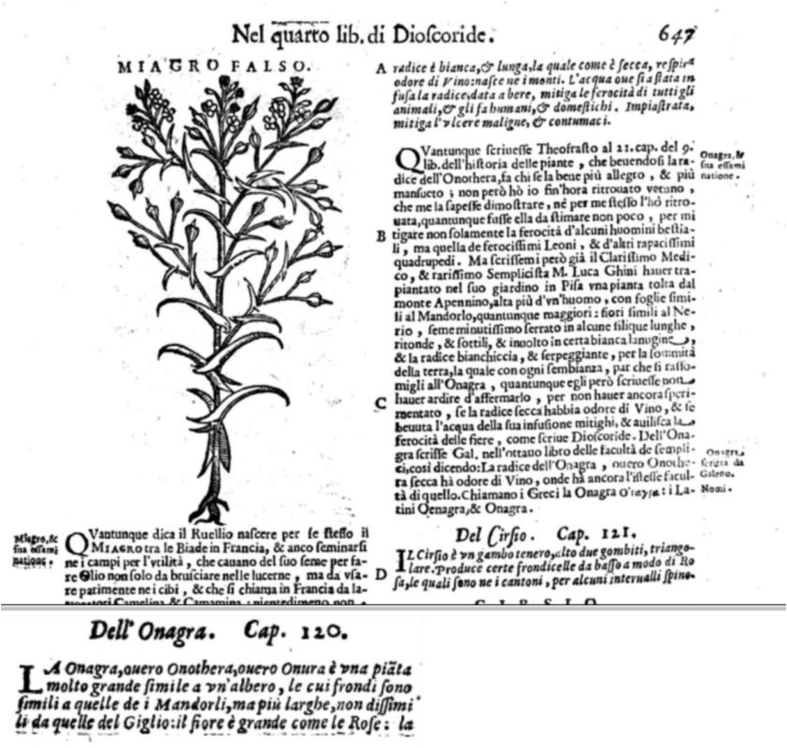
Chapter on *Onagra* in Mattioli (1645). NB: image does not belong to *Onagra* but to "Miagro falso".

It is interesting that in one of the later reprint of his commentaries, on the same page as *Onagra* a picture of another plant is provided, which may appear similar to *Epilobium* species to the untrained and careless eye who may also not notice that image does not belong to Onagra but to "Miagro falso" (Fig. 7).

**Fig. 8:**
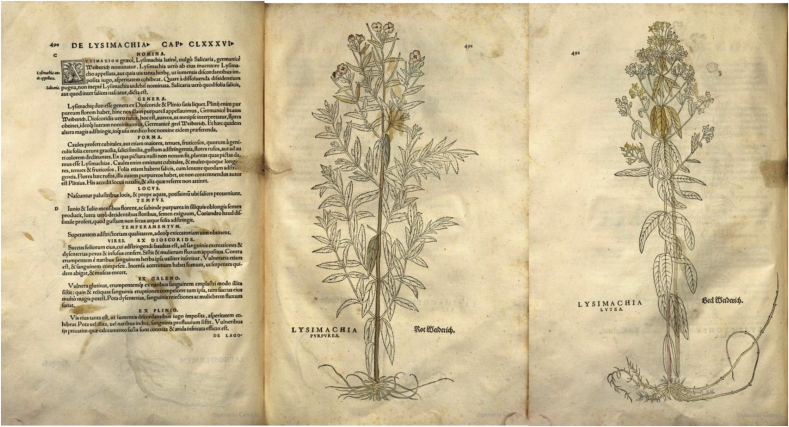
Pages of Fuchs (1542) later related to *E. angustifolium* (or *E. hirsutum* – which is most likely depicted).

While Leonhardt Fuchs (1501–1566), the contemporary of Mattioli, mentions *Lysimachia* in his book published few years before Mattioli and provides an illustration resembling *Epilobium hirsutum*, he does not mention *Onagra* (Fig. 8). Fuchs also describes the “*Lysimachia*” properties previously reported by Dioscorides, Galen and Pliny the Elder.

Therefore, it is most likely just a series of misinterpretations and the ambivalent name that created the perception of *E. angustifolium* as a potent medicine worthy of investigation at the beginning of the 21st century. We can see how the ancient texts, although indirectly, have affected people who were not even aware of their existence (cf Leonti, 2011). While good standards of practice have been developed for modern ethnopharmacology studies (Heinrich et al., 2018; Weckerle et al., 2018), there are no such standards for the interpretation of historical data. One of the reasons for misinterpretation today is popularization: as there are no standards for popular herbals, they combine both official and local uses of medicinal plants. The latter are often referred to as “popular use”, with which the authors absolve themselves from the burden of responsibility.

## 4 Conclusions

We can conclude that traditional uses of *E. angustifolium* were related to the treatment of wounds and skin diseases, fever, pain (headache, sore throat, childbirth), and abdominal-related problems (constipation, stomach ache) and intestinal bleeding. A few more uses were based on the similarity principle. The main theme, however, is the fragmentation of use and its lack of consistency apart from wounds and skin diseases.

It could simply be a coincidence that Maria Treben's suggestions, which gave rise to the advanced research on *E. angustifolium* in Europe, are so similar to the ones originating from the American herbals from the end of the 19th century. However, we have seen that the currently very popular and highly researched *E. angustifolium* had its moment of fame in the second half of the 20th century in North America, most likely due to being mistaken for a medicinal plant through the inaccurate interpretation of ancient sources. Nevertheless, its uses, despite being both widely adopted by practicing herbalists and official medicine in America, did not remain in use for long, and the temporal popularity resulted in only a few records among American Indians. Also the published doubts in its usefulness (and maybe even side-effects contrary to the suggestions, caused by over-consumption) might have contributed to the decrease of popularity.

It is entirely understandable that earlier authors made mistakes during the transcription of sources, simply by mixing up the card files or misinterpreting handwritten notes. Yet today, when historical sources are often digitized and made freely available on the Internet, it would be worth the effort of authors who attempt to interpret historical uses of plants to try to consult the original source (if still available) so as to not create the repeated chain of ghost data. Historical ethnobotanical investigations could help to avoid creating repeating waves of popularity of plants that have already been tried for certain diseases and later abandoned as not fully effective. The interdisciplinarity encoded in ethnobotany allows seeing beyond one field of study, encompassing different methodological and conceptual approaches, assessing not only facts, but also the context and the origin of every specific piece of information, thus testing its reliability within the specific source and against the wider background of international use.

There is, of course, a chance that *E. angustifolium* could also finally be proven to be clinically safe and cost-effective for treating benign prostatic hyperplasia, but this has not yet happened despite recent intensive research. Documented traditional use would suggest investigating the dermatological, intestinal anti-hemorrhagic and pain inhibiting properties of this plant, if any.

## Authors’ contributions

RS and RK designed the study; RS drafted the manuscript; RK, RS, VK, OB, JP, NS, BP, AS, NK conducted the field research; RS and RK conducted historical research; SM, GM, VK, NK, AI and AS contributed to historical research providing access, translating or interpreting some sources; RS, RK, SM and LK contributed to the discussion. All authors read and approved the final manuscript.
